# Rapid Vascular Responses to Anthrax Lethal Toxin in Mice Containing a Segment of Chromosome 11 from the CAST/Ei Strain on a C57BL/6 Genetic Background

**DOI:** 10.1371/journal.pone.0040126

**Published:** 2012-07-05

**Authors:** Kelsey J. Weigel, Laura Rues, Edward J. Doyle, Cassandra L. Buchheit, John G. Wood, Ryan J. Gallagher, Laura E. Kelly, Jeffrey D. Radel, Kenneth A. Bradley, Steven M. LeVine

**Affiliations:** 1 Department of Molecular and Integrative Physiology, University of Kansas Medical Center, Kansas City, Kansas, United States of America; 2 Rockhurst University, Kansas City, Missouri, United States of America; 3 Department of Surgery, University of Kansas Medical Center, Kansas City, Kansas, United States of America; 4 Department of Occupational Therapy Education, University of Kansas Medical Center, Kansas City, Kansas, United States of America; 5 Department of Microbiology, Immunology & Molecular Genetics, University of California Los Angeles, Los Angeles, California, United States of America; Duke University Medical Center, United States of America

## Abstract

Host allelic variation controls the response to *B. anthracis* and the disease course of anthrax. Mouse strains with macrophages that are responsive to anthrax lethal toxin (LT) show resistance to infection while mouse strains with LT non-responsive macrophages succumb more readily. B6.CAST.11M mice have a region of chromosome 11 from the CAST/Ei strain (a LT responsive strain) introgressed onto a LT non-responsive C57BL/6J genetic background. Previously, B6.CAST.11M mice were found to exhibit a rapid inflammatory reaction to LT termed the early response phenotype (ERP), and displayed greater resistance to *B. anthracis* infection compared to C57BL/6J mice. Several ERP features (e.g., bloat, hypothermia, labored breathing, dilated pinnae vessels) suggested vascular involvement. To test this, Evan’s blue was used to assess vessel leakage and intravital microscopy was used to monitor microvascular blood flow. Increased vascular leakage was observed in lungs of B6.CAST.11M mice compared to C57BL/6J mice 1 hour after systemic administration of LT. Capillary blood flow was reduced in the small intestine mesentery without concomitant leukocyte emigration following systemic or topical application of LT, the latter suggesting a localized tissue mechanism in this response. Since LT activates the Nlrp1b inflammasome in B6.CAST.11M mice, the roles of inflammasome products, IL-1β and IL-18, were examined. Topical application to the mesentery of IL-1β but not IL-18 revealed pronounced slowing of blood flow in B6.CAST.11M mice that was not present in C57BL/6J mice. A neutralizing anti-IL-1β antibody suppressed the slowing of blood flow induced by LT, indicating a role for IL-1β in the response. Besides allelic differences controlling Nlrp1b inflammasome activation by LT observed previously, evidence presented here suggests that an additional genetic determinant(s) could regulate the vascular response to IL-1β. These results demonstrate that vessel leakage and alterations to blood flow are part of the rapid response in mice resistant to *B. anthracis* infection.

## Introduction

Anthrax is a potentially lethal infection caused by *B. anthracis.* Anthrax lethal toxin (LT) is a major virulence factor that is comprised of protective antigen (PA) and lethal factor (LF) [1]. LT has dual effects on the host; it blunts the host immune response enabling the infection to become established, and it induces pathological changes similar to those observed in end stage toxemic disease following spore infection [2]–[8]. PA binds to anthrax toxin receptors, capillary morphogenesis gene 2 and/or tumor endothelial marker 8 [9], and is activated by serum proteases and/or cellular furin, which cleave PA into two fragments, PA_20_ (20 kDa) and PA_63_ (63 kDa) [10], [11]. PA_63_ self-associates into an oligomer [12], [13] and binds up to four molecules of LF before internalization [13], [14]. Once internalized, the complex changes conformation into an integral membrane pore that enables LF to pass into the cytosol [15], [16]. Inside the cell, LF operates as a zinc-dependent metalloproteinase that inactivates mitogen-activated protein kinase kinases (MKKs) thereby disturbing cellular signaling processes [17]–[19].

Susceptibility to infection is dependent on the interplay between the infectious agent and the host immune response. Following exposure to microbial molecules, a number of signaling mechanisms are initiated, such as the MKK pathway, which promote an inflammatory response that counters infection [20]. Inhibition of the MKK pathway by LF serves to block the immune response providing an advantage for *B. anthracis* survival [8]. However, genetic variation has enabled some hosts to make use of countermeasures that bypass MKK inhibition. For example, macrophages from 129S1 and CAST/Ei mice are responsive to LT, i.e., they undergo pyroptosis, while macrophages from other strains, e.g., C57BL/6J, are non-responsive. This variable response is due to allelic variation in the *Nlrp1b* gene [21], which is found within a LT sensitivity locus, *Ltxs1*, on chromosome 11. *Nlrp1b* is a member of the Nod-like receptor family, and activation of *Nlrp1b* prompts a cascade of events leading to inflammasome activation in macrophages from LT responsive animals [21], [22]. Inflammasome activity is a central response induced by exposure to other infectious agents, including *Salmonella typhimurium, Francisella tularensis, Listeria monoctyogenes* and *Staphylococcus aureus* [23].

*Nlrp1b* signaling results in the recruitment of caspase-1 to the inflammasome complex whereby it becomes activated, resulting in the cleavage of pro-IL-18 and pro-IL-1β into their mature forms which are released to further the immune response [24]. IL-18 activates neutrophils [25], [26], induces cytotoxicity of natural killer (NK) cells, increases the cytotoxicity of NK-T cells [27], and initiates the production of interferon-γ by activated NK and NK-T cells [28]–[31]. IL-1β triggers activation of nuclear factor κB (NF-κB) and MKKs [32], [33], and it initiates systemic and local inflammatory responses that facilitate the recruitment of inflammatory cells to the site of infection [34]–[36]. Transgenic mice with a sensitive 129S1 *Nlrp1b* allele on resistant C57BL/6J background (B6 *^Nlrp1b^* ^(129S1)^) exhibit heightened serum levels of IL-1β compared to C57BL/6J mice in response to LT [7]. An increased production of IL-1β in response to LT was also observed in congenic mice that have a segment of chromosome 11 from the CAST/Ei strain introgressed onto a C57BL/6J background (B6.CAST.11M) [37]. Both B6 *^Nlrp1b^* ^(129S1)^ and B6.CAST.11M strains exhibit an early response phenotype (ERP) that typically has an initial presentation 0.5–1 h following LT exposure and includes hypothermia, ataxia, bloat, loose feces, abdominal breathing and/or dilated vessels on pinnae [7], [37]. The ERP and increased responsivity of macrophages to LT are associated with increased resistance to challenge with *B. anthracis*, and signaling through the IL-1 receptor is required for the increased resistance in B6 *^Nlrp1b^* ^(129S1)^ mice [37]. Other studies have also shown this inverse relationship, i.e., mice with macrophages sensitive to LT are more resistant to *B. anthracis* infection [38]–[41]. Although the presence of an LT-responsive allele of *Nlrp1b* is sufficient to initiate a mild ERP [7], the greater genetic variation present in B6.CAST. 11M mice drives a more severe ERP and increased resistance to *B.* *anthracis* infection [37]. Further, B6.CAST.11M mice display an increased inflammatory response to muramyl dipeptide (MDP) plus lipopolysaccharide (LPS), suggesting that allelic variation on chromosome 11 loci other than *Nlrp1b* controls responsiveness to inflammatory mediators [37]. Thus, the utilization of B6.CAST.11 mice will enable the study of enhanced inflammatory responses that are potentially under the regulation of multiple genetic determinants on chromosome 11.

Because ERP is associated with resistance to *B. anthracis* infection, we wished to explore underlying mechanisms. Many of the characteristic features of the ERP suggest involvement of blood vessels. It has long been known that the introduction of bacteria to a mammalian host induces vascular responses. Vascular changes include increased permeability of capillaries, accumulation and diapedesis of leukocytes at vessels, and obstruction of vessels [42], [43]. In response to some bacteria or bacterial toxins, increases in vessel permeability can occur relatively rapidly [42], [43]. In addition, platelet aggregation and slowing of capillary blood flow occur within the first few hours of infection and act to limit the dissemination of bacteria [44]. In the present study, we examined whether vessels from B6.CAST.11M mice have increased permeability in response to LT compared to vessels from control animals. In addition, we employed intravital microscopy to study microvascular changes in response to LT, or downstream mediators, in real time in an *in vivo* setting.

## Results

### Vessel Leakage is Increased in the Lungs of B6.CAST.11M Mice Compared to C57BL/6J Mice Given LT

**Figure 1:**
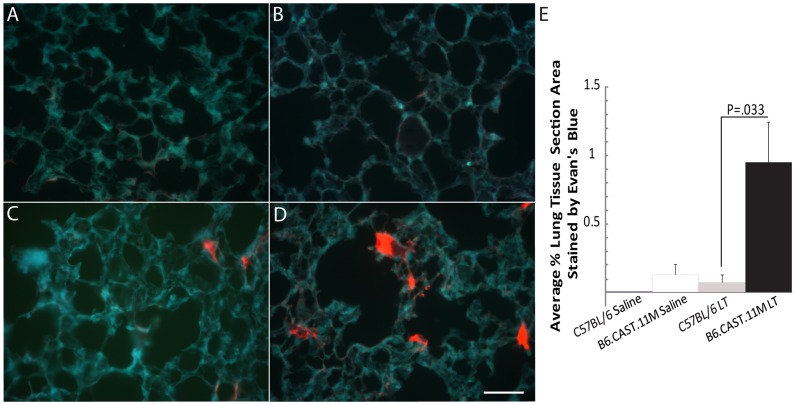
Evan’s blue leakage in lungs of B6.CAST.11M and C57BL/6J mice. **A**) C57BL/6J mice given saline, **B**) B6.CAST.11M mice given saline, **C**) C57BL/6J mice given LT, and **D**) B6.CAST.11M mice given LT by i.p. injection were sacrificed 1 h post administration. Evan’s Blue fluoresces bright red under UV light. Evan’s Blue was more wide spread in B6.CAST.11M LT mice than in other groups. Scale bar  = 50 µm. **E**) The average percent lung tissue section area stained with Evan’s Blue. Error bars represent standard error of the mean. C57BL/6J mice given saline (N = 5), B6.CAST.11M mice given saline (N = 10), C57BL/6J mice given LT (N = 8), B6.CAST.11M mice given LT (N = 13).

Labored breathing is a prominent feature of the ERP and we predicted that pulmonary vascular leakage would be associated with this clinical sign. Thus, Evan’s blue was administered to mice 30 min after receiving LT and mice were sacrificed 30 min later. All animals included in the study were confirmed to have successful uptake of Evan’s blue into the systemic circulation. Evan’s blue was observed within capillaries and/or extravasated from vessels. Quantitation of extravasated Evan’s blue revealed that B6.CAST.11M mice given LT had greater pulmonary vascular leakage than for C57BL/6J mice given LT, p = 0.033 (Fig. 1).

### Blood Flow is Reduced in B6.CAST.11M Mice Compared to C57BL/6J Mice Following Systemic Administration of LT

**Figure 2:**
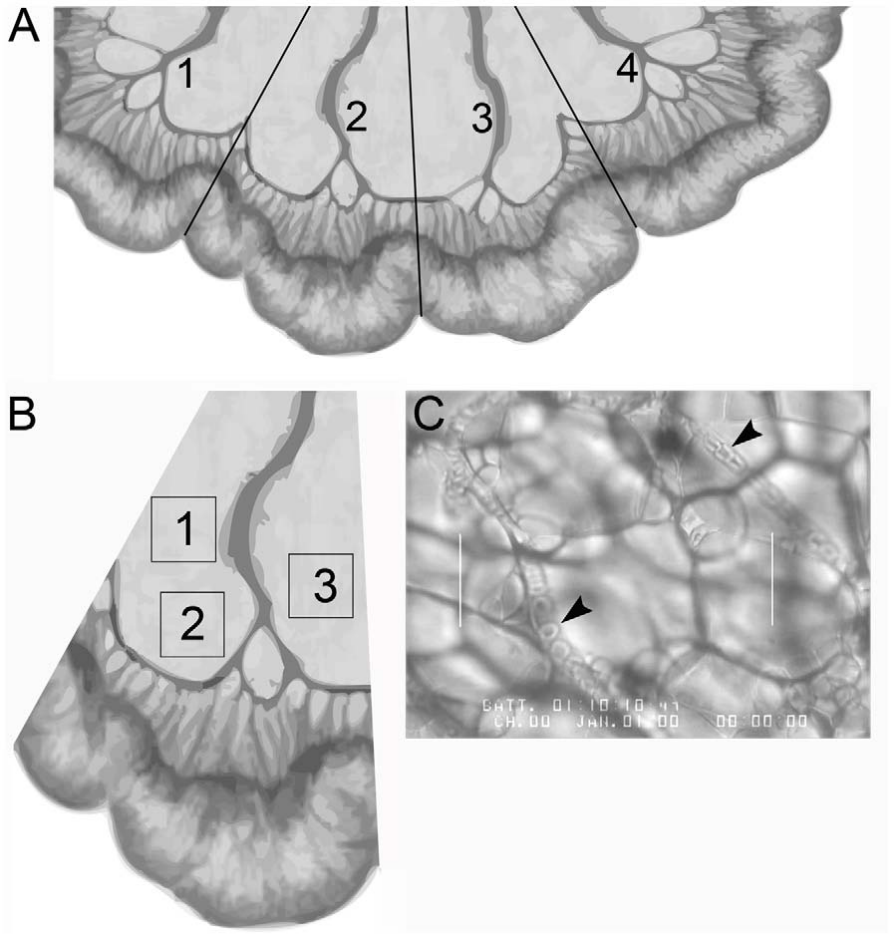
Method used to measure RBC velocity within capillary beds. In order to obtain an accurate portrayal of the changes in the mesenteric blood flow, multiple areas of mesentery were sampled. The various mesenteric areas were analyzed in sequential fashion, and then the evaluation process was repeated multiple times so that the changes in blood flow could be monitored over time in the living animal. **A**) Four intestinal loops were analyzed per mouse for systemic studies. **B**) One loop from A is shown. Three areas per intestinal loop (boxes 1, 2, 3) were observed within a viewing interval. Each viewing interval consisted of 6 min of data collection over the 3 areas (2 min/area). This was followed by a 10 min period before repeating the process in the next loop. For systemic studies, four loops were viewed in sequence and this sequence was repeated 3 times. Thus, there were a total of 12 viewing intervals (4 intestinal loops × 3 repetitions/loop). Since 3 areas/loop were analyzed for each viewing interval this resulted in 36 areas viewed (3 areas/loop × 12 viewing intervals) per experiment. For topical studies, one loop was viewed for 7 viewing intervals. Thus, there were a total of 21 areas viewed (3 areas/loop × 7 viewing intervals) per experiment. **C**) Representative intravital microscopic image (still video frame) from one area depicted in panel B. Many frames were scanned and recorded within the 2 min/area viewing interval. The frame with the maximum pathology was selected for each area to calculate and analyze. Arrowheads point to RBCs in capillaries. The distance between the two parallel lines is 100 µm. The diagrams in panels A and B were extensively adapted from a figure in the book by Gray H (1918) Anatomy of the Human Body [90].

Vascular leakage induces coagulation, which in turn can cause blood flow to be sluggish [45]–[47]. In order to address whether systemic administration of LT leads to alterations in blood flow, we employed intravital microscopy, which enables blood flow to be monitored over time in living animals. The mesentery is particularly well suited for intravital microscopic analysis due to its relatively transparent properties and its accessibility. Thus, blood flow was analyzed in the mesentery of B6.CAST.11M and C57BL/6J mice given systemic administration of LT (Fig 2).

**Figure 3:**
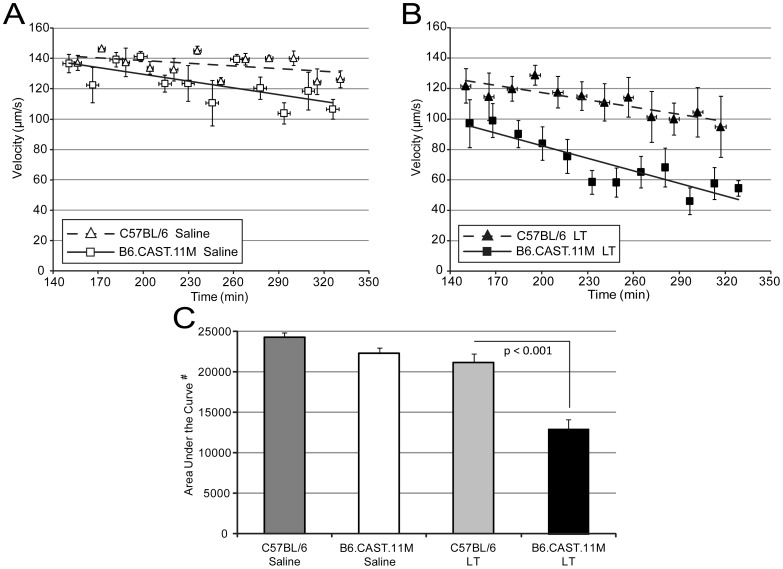
Systemic LT reduces RBC velocity in mesentery capillaries in B6.CAST.11M mice. RBC velocities within capillaries were determined as described in Fig 2 for animals given an i.p. injection of **A**) saline or **B**) LT. Points represent the average velocity of the RBCs in the three areas within each loop at the indicated times (12 points  =  4 intestinal loops × 3 repetitions/loop). Regression lines were calculated from these 12 points/mouse group. Error bars represent standard error of the mean. **C**) The area under regression lines for animals given saline (corresponding to panel A) or LT (corresponding to panels B) (# indicates arbitrary units). Error bars represent standard error of the mean. Area under the curve is considered an informative measure of overall disease activity [91]. The area under regression lines was less in B6.CAST.11M mice receiving LT compared to C57BL/6J mice receiving LT (p<0.001) or B6.CAST.11M mice receiving saline (p<0.001). The area under the curve was not statistically different between B6.CAST.11M and C57BL/6J mice given saline. (C57BL/6J given saline, n = 4; B6.CAST.11M given saline, n = 6; C57BL/6J mice given LT, n = 5; and B6.CAST.11M given LT, n = 8).

B6.CAST.11M but not C57BL/6J mice typically displayed initial signs of the ERP by 1 h in response to i.p. administration of LT, consistent with previous findings [37]. Following the ∼1 h observation period, animals were prepared for analysis by intravital microscopy. Since the ERP has a strong presentation in B6.CAST.11M mice during the first 7 h following LT administration [48], observations were made within this time period. Evidence of rolling or adherence of leukocytes along vessels in the mesentery was not readily apparent in B6.CAST.11M or C57BL/6J mice receiving saline or LT (not shown). RBC velocities in capillaries were similar between B6.CAST.11M and C57BL/6J mice receiving saline (Fig. 3A) but were slower in B6.CAST.11M mice receiving LT compared to C57BL/6J mice receiving LT (Fig. 3B). The slopes of regression lines between B6.CAST.11M mice and C57BL/6J mice receiving LT were not significantly different, but the area under regression lines, which is a measure of total blood flow over the duration of the collection of data, was less in B6.CAST.11M mice receiving LT compared to C57BL/6J mice receiving LT (p<0.001) (Fig. 3C). Velocity measurements reflected averaged values of blood flow within capillaries (Fig. 2), with some capillaries exhibiting normal or reduced blood flow while others were completely stopped and/or coagulated in B6.CAST.11M mice given LT. A difference in area under the curve between B6.CAST.11M and C57BL/6J mice receiving systemic LT despite an absence of a difference in slopes suggested that the changes in slope occurred prior to the timing of the intravital microscopic observations. In order to test this possibility, LT was topically applied and immediately followed by intravital measurements of blood flow (see below).

### Rapid Reduction of Blood Flow in B6.CAST.11M Mice, but not C57BL/6J Mice, Following Topical Application of LT

**Figure 4:**
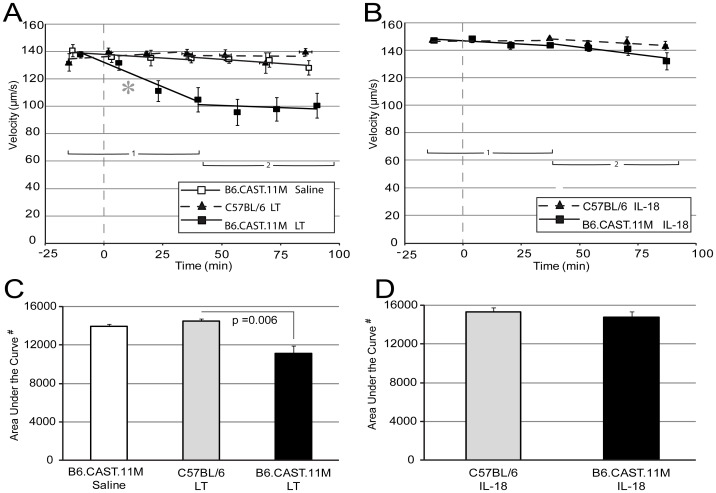
Effects of topical LT or IL-18 on RBC velocities in mesentery capillaries. Dashed vertical lines represents initial application of topical agent (prior to agent is topical saline). Points represent the average velocity of RBCs within the three areas viewed at the indicated times (see Methods and Fig 2 for details). Due to the change in slopes over time, two regression lines were calculated. The first regression line uses the first four viewing intervals. The second line uses the fourth through seventh viewing intervals. Error bars represent standard error of the mean. **A**) The slope of regression line 1 (asterisk), but not regression line 2, for RBC velocities in capillaries was more negative in B6.CAST.11M mice given topical application of LT compared to C57BL/6J mice given LT (p = 0.008). B6.CAST.11M LT group n = 8, and B6.CAST.11M saline and C57BL/6J LT groups n = 5. **B**) B6.CAST.11M and C57BL/6J mice given topical application of IL-18. RBC velocities in capillaries remained relatively unchanged over time and there were no differences for RBC velocities between B6.CAST.11M and C57BL/6J mice given topical application of IL-18. Both animal groups, n = 5. **C**) The area under the curve (corresponding to panel A) was less in B6.CAST.11M mice given topical application of LT compared to C57BL/6J mice given topical application of LT (p = 0.006) (# indicates arbitrary units). **D**) The area under the curve (corresponding to panel B) was comparable between C57BL/6J and B6.CAST.11M mice given topical application of IL-18.

In order to investigate rapid responses to LT and further determine whether the slowing blood flow response observed in the B6.CAST.11M mice given systemic LT could be elicited at the local tissue level, naïve mice prepped for intravital microscopy were challenged with topical administration of LT or saline to the mesentery. As observed for systemic LT, rolling or adherence of leukocytes along vessels were not readily apparent in B6.CAST.11M and C57BL/6J mice receiving saline or LT (not shown). No substantial changes in the RBC velocities in capillaries were observed over time in B6.CAST.11M mice receiving saline or C57BL/6J mice receiving LT (Fig. 4A). However, B6.CAST.11M mice receiving LT displayed a rapid slowing of RBC velocity followed by a leveling off phase (Fig. 4A). Since the measurements reflected averaged values of blood flow, blood flow stoppage in capillaries was often observed in the B6.CAST.11M mice given topical application of LT. The slope revealing the initial change in RBC velocity in capillaries from B6.CAST.11M mice receiving LT was steeper, i.e., more negative, than the C57BL/6J mice receiving LT (p = 0.008), but there was no difference in the second plateau phase between these groups (Fig. 4A). The area under the curve was also less in B6.CAST.11M mice given topical application of LT compared to C57BL/6J mice given LT (p = 0.006) (Fig. 4C). Of note, the plateau of velocities (phase 2) in capillaries from B6.CAST.11M mice receiving topical LT (Fig. 4A) likely accounts for the lack of a difference in slopes of RBC capillary velocities between B6.CAST.11M and C57BL/6J mice receiving systemic LT as the timing of data collection in the systemic studies was delayed following LT administration (Fig. 3B).

### Topical Application of IL-1β, but not IL-18, Induces a Rapid Slowing of Blood Flow in B6.CAST.11M Mice but not C57BL/6J Mice

**Figure 5:**
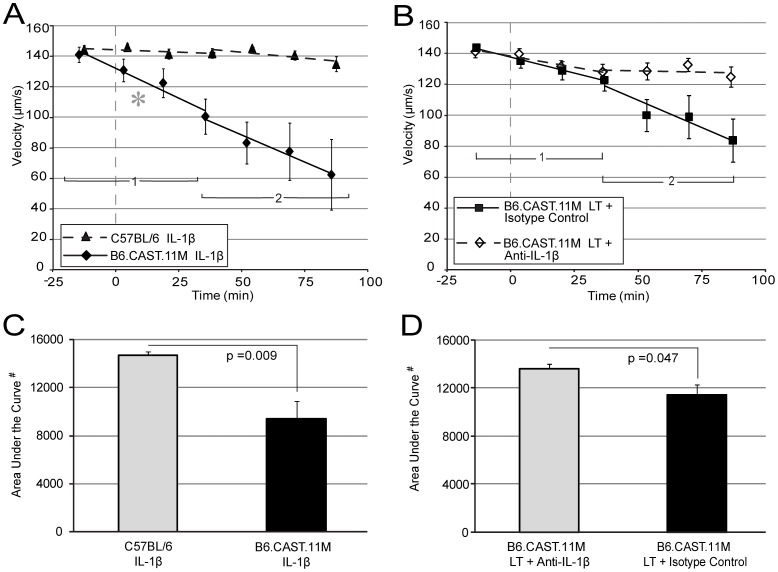
Role of IL-1β on RBC velocities in capillaries. Vertical dashed lines represent initial application of topical agent (prior to agent is topical saline). Points represent the average velocity of RBCs within the three areas viewed at the indicated times (see Methods and Fig 2 for details). Similar to Fig. 4A,B, two regression lines were calculated. The first regression line uses the first four viewing intervals. The second line uses the fourth through seventh viewing intervals. Error bars represent standard error of the mean. **A**) B6.CAST.11M mice given a topical application of IL-1β had a more negative slope of RBC velocities in capillaries for phase 1 (asterisk), but not phase 2, compared to C57BL/6J mice given IL-1β. Each animal group had n = 5; slope of regression line phase 1, p = 0.002. **B**) B6.CAST.11M mice given a topical application of rat anti-mouse IL-1β plus LT had a tendency for a less negative slope in phase 2 compared to B6.CAST.11M receiving rat IgG1 isotype control antibody plus LT, but did not reach significance (p = 0.075). Each animal groups, n = 5. **C**) B6.CAST.11M mice given a topical application of IL-1β had a smaller area under regression line compared to C57BL/6J mice given IL-1β (p = 0.009) (corresponding to panel A) (# indicates arbitrary units). **D**) B6.CAST.11M mice given a topical application of rat anti-mouse IL-1β plus LT had a larger area under the regression line compared to B6.CAST.11M receiving rat IgG1 isotype control antibody plus LT (p = 0.047) (corresponding to panel B).

Activation of the inflammasome complex results in the production of mature forms of IL-18 and IL-1β. Since LT induces inflammasome activation in B6.CAST.11M mice [37], we investigated whether either of these cytokines could cause changes in RBC velocities. IL-18 did not induce an appreciable change in RBC velocities in capillaries in either B6.CAST.11M or C57BL/6J mice (Fig. 4B,D). However, topical application of IL-1β resulted in a steady decline in RBC velocities in capillaries over the entire course of the experiment in B6.CAST.11M mice, but not C57BL/6J mice (Fig. 5A). Since the measurements reflected averaged values of blood flow, blood flow stoppage in capillaries was often observed in the B6.CAST.11M mice given topical application of IL-1 β. The B6.CAST.11M mice had a more negative slope in the capillary phase 1 (p = 0.002) (Fig. 5A) and a smaller area under regression lines of the RBC velocities compared to C57BL/6J mice given IL-1β (p = 0.009) (Fig. 5C). Unlike the topical LT administration to B6.CAST.11M mice (Fig. 4A), no leveling off was evident following the initial response (phase 1) when IL-1β was applied, which could be a consequence of the continued application of IL-1β whereas IL-1β produced in response to LT-induced inflammasome activation would be expected to be released in a bolus associated with rapid macrophage pyroptosis.

### IL-1β Produced in Response to LT Contributes to the Reduction of Blood Flow in B6.CAST.11M Mice

Responsiveness to IL-1β demonstrated above indicates that this cytokine is sufficient to induce strain-specific vascular changes. However, many inflammatory mediators, in addition to IL-1β and IL-18, are released following Nlrp1b-dependent pyroptosis [7], [37]. To test whether IL-1β is necessary for LT induced RBC slowing, topical application of anti-IL-1β antibody was coadministered with LT to B6.CAST.11M mice. These data were compared to the topical application of an IgG_1_ isotype control antibody co-administered with LT to B6.CAST.11M mice. Although the slopes were not different (Fig. 5B), overall, the RBC velocities were slower in capillaries in B6.CAST.11M mice receiving IgG_1_ isotype control plus LT compared to B6.CAST.11M mice receiving anti-IL-1β plus LT (area under the regression lines for capillaries, p = 0.047) (Fig. 5D). This decrease in area under the regression lines was due to the former group having a greater reduction in phase 2 (Fig. 5B).

## Discussion

Microvascular changes are a cardinal feature in response to bacterial infection. These events (e.g., increased capillary permeability and “stickiness” of the endothelium) can occur rapidly after the inoculation of bacteria, and depending on the type of bacteria, there is often a resolution back to a near normal state at ∼1 h followed by a second response that can be greater than the first [42], [43]. The ERP in B6.CAST.11M mice typically begins 0.5–1 h after intraperitoneal LT administration [37]. It too is followed by a resolution phase and a secondary response, albeit with delayed timing relative to the inoculation of bacteria [37]. Many features of the ERP (e.g. bloat, dilated vessels on pinnae, difficulty breathing, hypothermia) were predicted to occur in response to changes at the vascular level. Studies utilizing Evan’s blue revealed vessel leakage in the lung of B6.CAST.11M mice exposed to LT but not in control mice (Fig. 1). Vessel leakage or pleural effusion has been observed in other animal models following LT exposure indicating that other bacterial products are not required for this process [3], [6], [49]–[51]. However, no mouse model was described to have vascular permeability changes within the early time period as seen for B6.CAST.11M mice except for an intradermal LT model [52], which is more relevant to cutaneous anthrax. In this latter study [52], most responding mouse strains contain a sensitive *Nlrp1b* allele [21], which results in inflammasome activation leading to IL-1β production [24].

Vascular leakage can lead to coagulation, which in turn can affect blood flow [46], [53]–[55]. Additionally, vascular leakage leads to tissue edema, which can also reduce blood flow [56]. A reduction in capillary blood flow, e.g., due to coagulation, functions to limit the spread of bacteria [44]. In order to address whether vessel leakage is associated with reduced blood flow during the host inflammatory response to LT, we employed intravital microscopy for real time examination of the vasculature in mice. Using this technique, we observed a pronounced slowing of the blood flow in capillaries of the small intestinal mesentery in B6.CAST.11M animals after exposure to systemic LT administration (Fig. 3B,C). Slowing of blood flow was also observed following topical application of LT (Fig. 4A,C) suggesting that direct action by LT on the vasculature and/or mediators produced at the local tissue level, e.g., by tissue myeloid cells, were sufficient to induce this change. The lower average values of blood flow following systemic or topical LT in B6.CAST.11M mice reflect that many capillaries had blood flow stoppage or coagulation.

Mediators that could be responsible for inducing vascular changes include proinflammatory cytokines. Indeed serum levels of 23 cytokines increased in association with the ERP in B6.CAST.11M animals [37]. The ERP has been linked to a genetic locus within a critical region in chromosome 11 that accounts for a high level of responsivity to LT and resistance to *B.* *anthracis* [37]. This region encodes numerous genes associated with immune function including the inflammasome component *Nlrp1b*. Allelic variation of *Nlrp1b* controls LT-sensitivity of macrophages by determining inflammasome activation and subsequent caspase-1 mediated processing of pro-IL-1β and pro-IL-18 [21], [37]. When applied directly to the mesentery, IL-1β induced blood flow slowing in B6.CAST.11M mice (Fig. 5A,C) and contributed to the slowing of blood flow following LT exposure in B6.CAST.11M mice (Fig. 5D). C57BL/6J mice did not show altered blood flow in response to topical application of IL-1β (Fig. 5A), and they did not display an ERP in response to systemic IL-1β [37], indicating that a genetic determinant(s) that is present in B6.CAST.11M mice is responsible for these responses. The finding that transgenic mice with a responsive 129S1 allele of *Nlrp1b* on a C57BL/6J background display a moderate ERP when challenged with LT [7], [37] indicates that a responsive *Nlrp1b* allele is sufficient to initiate this phenotype. However, in addition to *Nlrp1b*, twenty-five candidate genes have been identified that could contribute to the significantly more pronounced ERP observed in B6.CAST.11M mice [37] and one or more of these could control the response to IL-1β. Unlike the response to IL-1β, application of IL-18, another inflammasome product, had no effect on the slowing of blood flow in capillaries (Fig. 4B,D).

There is precedence for closely spaced genetic determinants on chromosome 11 regulating a response to LT in mice; three separate quantitative trait loci within an ∼12 cM region of chromosome 11 were found to influence the survival of mice exposed to LT [57]. Other published findings also support a role for an additional genetic determinant(s), other than *Nlrp1b*, on chromosome 11 contributing to inflammatory responses [37]. For example, ataxia and hypothermia are enhanced in B6.CAST.11M mice compared to C57BL/6J mice in response to MDP + LPS challenge [37], yet the response to this challenge is not thought to be controlled by the *Nlrp1b* locus. Furthermore, F1 mice (offspring from B6.CAST.11M and C57BL/6J mice) displayed a diminutive ERP from that observed in B6.CAST.11M mice [37], yet the responsive *Nlrp1b* allele is thought to act in a dominant manner [58]. However, the in vitro assay used to make this determination could have lacked sensitivity to detect an intermediate response. Finally, no differences in LT-induced IL-1β release or LT dose-dependent pyroptosis were observed in bone marrow derived macrophages from B6 *^Nlrp1b^* ^(129S1)^ or B6.CAST.11M mice [37]. It is possible that a regulatory feedback loop acting through *Nlrp1b* could account for one or more of the findings. If this were the case, it would indicate that allelic variation in *Nlrp1b* could enable further inflammasome activation in response to IL-1β or downstream mediator. Alternatively, an additional genetic determinant(s) could account for the differential response to IL-1β observed between B6.CAST.11M and C57BL/6J mice (Fig. 5A,C).

Cytokine production in response to infection or inflammation triggers coagulation via the extrinsic pathway [59], [60], i.e., complexation of tissue factor (TF) to factor VII is an early step in the initiation of the blood coagulation cascade [59]–[61] leading to platelet activation [62], [63]. These events can result in sludging, or reduced blood flow, in the microvasculature. TF is produced by monocytes and macrophages and it is possible that cytokines, e.g., IL-1β and TNFα, also induce endothelial cell expression of TF, but whether this occurs in vivo is uncertain [59]. LT does not induce tissue factor (TF) in cultured human umbilical vein endothelial cells and it suppresses LPS-induced TF in these cells [64]. However, the effect of LT on TF expression on monocytes/macrophages, endothelial, and other cell types in B6.CAST.11M mice is unknown. During early stage anthrax or LT intoxication in B6.CAST.11M mice, the elevated level of IL-1β would be predicted to activate TF in various cell types [59], [65] assuming LT did not block this action.

Complement component 1, q subcomponent binding protein (C1qbp) and platelet-activating factor acetylhydrolase, isoform 1b, subunit 1 (Pafah1b1) were identified as potential modifier genes accounting for the ERP and/or resistance to *B. anthracis* in B6.CAST.11M mice [37], raising the possibility of their involvement in the slowing of blood flow. Neither these genes nor any of the other 25 candidate genes [37] were among the vessel associated genes up or down regulated in the lungs of A/J (or C57BL/6) mice at 6 h post exposure of LT compared to mutant LT (PA plus a mutated LF) [66]. Since the vascular changes and ERP occur rapidly in B6.CAST.11M mice, it is likely that up or down regulation of gene expression following LT administration was not a critical determinant for the observed phenotype, rather preexisting allelic differences or expression levels may be more relevant.

Leukocyte accumulation was not observed in the mesentery in response to topical or systemic LT or topical IL-1β in either B6.CAST.11M or C57BL/6J mice during the time frame of the experiments (∼5 h systemic application; ∼1.5 h topical application) using intravital microscopy. In a recent study, no leukocyte infiltration was observed in the lung of A/J or C57BL/6J mice that were sacrificed at 6 and 12 h post LT administration [66]. In another study, neutrophils did not infiltrate tissues following LT administration [6]. These results indicate that LT or downstream mediators are not sufficient for signaling leukocyte migration into tissue in mice in a rapid manner and that leukocyte accumulation is not responsible for reduced blood flow and vascular leakage in mice during this early period. Reduced vascular blood flow was also observed to be independent of leukocyte accumulation in a model of kidney infection [44] and vascular leakage can occur before infiltration of leukocytes [42], [43].

Vessel leakage and slowing of blood flow can now be added to the list of features that comprise the ERP, and LT, independent of other components from *B. anthracis*, is sufficient to induce these changes in B6.CAST.11M mice. How vessel leakage and slowing of blood flow might impact the disease process in response to a *B.* *anthracis* infection requires further investigation. Vessel leakage allows anti-microbial and blood components to access an infection site, while coagulation is thought to limit the spread of the infectious organism [44], [67], [68]. In addition, an ischemic state would result in tissue areas that contained vessels with a substantial reduction of blood flow. Ischemia could activate hypoxia-inducible factor-1α (HIF-1α) and downstream stress responses. These in turn could serve to protect cells in the affected tissue area against more extensive damage from the developing infection [69]–[71] and the protection could also counter side effects resulting from the ensuing immune response. However, these responses could be tissue specific. For example, bacterial infection of proximal tubules resulted in rapid hypoxia followed by reductions in blood flow before leukocytes infiltrated the tissue [44]. No HIF-1α was successfully detected in this model; rather tubule epithelial tissue sloughed off and limited bacterial dissemination [44]. Thus, ischemia and coagulation helped to isolate the infection and protect the host from fatal urosepesis [44].

Vessel leakage may be responsible for a number of the other features of the ERP. One characteristic of the ERP is labored abdominal breathing. A relationship between increased vascular permeability and increased breathing rates was noted after exposure to *B. anthracis* spores [51] and signs of illness such as breathing difficulty, including shallow, rapid breaths with increasing severity correlated to the time of vascular leakage and pulmonary edema after LT administration [3]. Thus, these studies support the notion that vessel leakage contributes to labored breathing, which is exhibited during the ERP. Bloat and dilated vessels on the pinnae may also be attributed to the vessel leakage.

Inflammasome activation and IL-1β production are important inflammatory responses in a number of pathogens or conditions such as Muckle-Wells syndrome, Ebola virus, Marburg virus, gout, pseudogout, Crohn’s disease, asbestosis, silicosis, Systemic Inflammatory Response Syndrome (SIRS), etc. [24], [45], [46], [72], [73]. In fact, transgenic mice expressing a 129S1 responsive *Nlrp1b* allele on a C57BL/6J background (B6 *^Nlrp1b^* ^(129S1)^ mice) had increased resistance to *B. anthracis* infection [7] and mild-to-moderate slowing of blood flow (not shown); however, if these mice were also made deficient for the IL-1 receptor, the resistance to *B.* *anthracis* was lost [37]. A requirement for IL-1 receptor signaling for resistance to *B. anthracis* infection was also observed in other studies [41], [74]. The strong response to IL-1β in B6.CAST.11M mice compared to an absence of a response in C57BL/6J mice (Fig. 4A) is in line with the greater resistance to infection in B6.CAST.11M mice compared to C57BL/6J mice [37]. This result could be explained by a model where a genetic determinant other than *Nlrp1b* generates a modified downstream response to IL-1 receptor signaling in B6.CAST.11M mice.

The role of hypothermia in B6.CAST.11M mice is unknown. In other models, e.g., MDP + LPS challenge, body temperature of the animal steadily decreases after IL-1β becomes activated [75]. However, this is response is mediated, at least in part, through TNFα [75]. Of note, B6.CAST.11M mice exposed to LT display a rapid increase in TNFα which is not seen in C57BL/6J mice exposed to LT [37]. Also, hypothermia has been observed in mice that have been primed with gram-positive *Propionibacterium acnes* and challenged with LPS or TNFα, and this response is linked to signs of hypercoagulation and SIRS [76]. In mice that experience SIRS, an early drop in body temperature is followed by a return to normal temperatures by 6–8 h [45] which is also similar to the profile of the ERP in B6.CAST.11M mice [37]. Hypothermia seems to act as a protective mechanism in delaying the mean time of death in animals exposed to anthrax toxin [77]. However, the role of hypothermia relative to resistance to infection remains to be determined.

Diseases that involve inflammasome activation and IL-1β production can result in vascular changes, e.g., SIRS patients experience increased microvascular permeability, platelet sludging, maldistribution of blood flow, and activation of the coagulation system, among other symptoms [45], [46]. Since IL-1β promotes vascular leakage [65] and slowing of blood flow (Fig. 5A,C), it would be tempting to speculate that IL-1β performs a similar role in these other diseases. However, these rapid effects were observed in B6.CAST.11M mice, but not in C57BL/6J mice, therefore caution must be applied when interpreting the role of IL-1β in inbred mouse models of infection and inflammasome mediated diseases. With that said, allelic variation provides insight into host response to infection and inflammation, and allelic variation does alter inflammatory responses in humans [78]–[80].

In summary, blood flow slowing and vessel leakage, but not leukocyte accumulation, are components of the ERP, which is present in B6.CAST.11M mice but not C57BL/6J mice following LT exposure. Vessel leakage is thought to contribute to slowing of blood flow through activation of the coagulation cascade and/or tissue edema. The inflammasome product IL-1β, but not IL-18, mediates slowing of blood flow. Further investigations are required to identify the roles of vessel leakage and reduced blood flow during the early stages of disease, but presumably they could limit the spread of infection and thus help confer resistance to disease.

## Materials and Methods

### Reagents and Mice

LT was comprised of PA and LF whose preparations were previously described [37]. Additional reagents used for intravital microscopy studies included recombinant mouse IL-1β/IL-1F2, rat anti-mouse IL-1β/IL-1F2 (IgG_1_ Antibody), rat IgG_1_ isotype control and recombinant mouse IL-18/IL-1F4 (R&D Systems, Inc., Minneapolis, MN). Evan’s blue was purchased from Sigma-Aldrich (St. Louis, MO).

All procedures involving the use of animals were approved by the Institutional Animal Care and Use Committee at the University of Kansas Medical Center. B6.CAST.11M mice were kindly provided by Drs. Aldons J. Lusis and Richard C. Davis (University of California at Los Angeles) and the generation of these mice has been described previously [81], [82]. B6.CAST.11M mice contain a CAST/Ei region in chromosome 11 (∼31.5 Mb to the terminus) on an otherwise C57BL/6J genetic background [82]. The study by Terra et al. [37] found that this strain develops an ERP in response to LT. C57BL/6J mice were purchased from Jackson Laboratory (Bar Harbor, ME).

### Evan’s Blue Administration

Previous studies established that i.p. administration of LT results in pathology in systemic organs [6], [83] and an i.p. injection of LT resulted in a similar profile of the ERP in sensitive mice compared to i.v. LT administration with the exception of the presentation of the ERP in former being slightly delayed [37]. Thus, i.p. injections were used to administer LT or saline, which was the vehicle control, to B6.CAST.11M or C57BL/6J mice [37]. A 200 µl i.p. injection of 0.22 µm filtered 10 mg/ml Evan’s blue in saline was administered 30 min post LT injection. Mice were sacrificed at 1 h post LT injection, and organs fixed in 10% formalin overnight. The following day, tissues were embedded in OCT (Histoprep) and frozen at -80°C. Lung tissue sections, 10 µm thick, were prepared in the coronal plane. Analyses were performed on 2 sections per animal that were separated by 50 µm.

### Stereological Analysis of Lung Sections

Each section of left lung was captured using a 2x objective under bright field illumination. An 11 × 9 grid was overlaid onto each image (ImageJ, http://rsbweb.nih.gov/ij/index.html). Four squares of each grid were selected randomly and the fluorescent image from each of these selected locations was captured using a 40x objective and UV2A (380–420 nm) cube (Nikon, NY, Melville).

### *Evan’s Blue Data Analysis*

Image analysis of each lung location was performed in a blinded manner using ImageJ. Split color channels were used to detect total tissue area or Evan’s blue fluorescent staining (red). The signal threshold was adjusted so that only the tissue area or fluorescent red staining area was revealed. Red fluorescent staining extending beyond the capillary boundaries was quantified while staining that traced only the capillaries was ignored. The average percent stained area of the four locations/section and 2 sections/animal was calculated for each subject.

### Intravital Microscopy Technique

For studies involving the systemic application of LT, B6.CAST.11M and C57BL/6J were given an i.p. injection of LT (15 µg PA and 7.5 µg LF per g body weight or titrated equivalent [37]) or saline, evaluated for clinical signs [7], [37] during the subsequent ∼1 h, anesthetized with urethane via i.m. injection(s) and prepared for intravital microscopy as previously described [84], [85]. In brief, the abdomen was opened along the midline using a radiocautery (Harvard Apparatus, Holliston, MA), and the animal was positioned on a plexiglass sheet with the small intestine carefully exteriorized over a glass coverslip. A Zeiss Axiovert inverted microscope was used to view the mesenteric microvasculature and a 40x objective was used for collection of images that were recorded on a Panasonic video cassette recorder with a time-date generator (Panasonic, Osaka, Japan). The mesentery was superfused throughout the experiment with warmed saline to keep the tissue moist, and a homeothermic blanket system was applied to the mouse (Harvard Apparatus, Holliston, MA). An optical Doppler velocimeter (Microcirculation Research Institute, College Station, TX) was used to measure centerline blood flow velocity within venules.

For studies involving the topical application of a test reagent(s), B6.CAST.11M and C57BL/6J mice were anesthetized with urethane, surgery was performed, and the small intestine was arranged and viewed as described above. Initially the mesentery was superfused with warmed saline. This was followed with the superfusion of a solution containing saline, LT (PA = 0.1875 µg/µl, LF = 0.09375 µg/µl), LT control (PA = 0.375 µg/µl in saline, or LF = 0.1875 µg/µl in saline), LT plus rat anti-mouse IL-1β (10 ng/µl), LT plus IgG_1_ (10 ng/µl), recombinant mouse IL-1β (0.0375 ng/µl), or recombinant mouse IL-18 (0.375 ng/µl). The concentration selected for IL-1β for intravital experiments was based off extrapolation of IL-1β dosages used in published work in whole mice [37], [86], [87]. The selection of a 10 fold increase in concentration for IL-18 was based on another study that utilized a 10 fold greater concentration of IL-18 compared to IL-1β [88] and the fact that IL-18 in the serum reaches an ∼10 fold higher concentration compared to IL-1β following MDP + LPS challenge [89]. Notably, MDP+LPS was also found to induce a differential response between C57BL/6J and B6.CAST.Ei mice [37]. Approximately 15 applications of 150 µl/application were superfused on the mesentery in each experiment. Blood flow velocities within venules were obtained as described above.

### Intravital Microscopy Data Collection

Capillaries were defined as having a diameter of approximately one red blood cell width. For systemic studies, capillary data was collected from four separate loops within the mesentery, each loop determined by the presence of an independent blood source (Fig. 2). Within each loop, three areas (each adjacent to a selected venule, velocity of ≤7 mm/sec) were viewed for 2 min each (Fig. 2). The viewing of the three areas over a 6 min period was a viewing interval. The four loops were viewed in sequence and this sequence was repeated three times. Thus, there were a total of 12 viewing intervals (4 intestinal loops × 3 repetitions/loop) resulting in 36 areas viewed (3 areas/loop × 12 viewing intervals) per experiment.

For topical studies, capillary data was collected from one loop within the mesentery. Within the loop, three areas (each adjacent to a selected venule, velocity of ≤7 mm/sec) were viewed per viewing interval as previously described. The loop was viewed a total of seven times, resulting in a total of 21 areas viewed (3 areas × 7 repetitions) per experiment.

### Intravital Microscopy Capillary Data Analysis

Video clips were viewed in Adobe Premiere (Adobe, San Jose, CA). During the review of video clips, the area of maximum pathology (e.g. greatest slowing, stopping and/or coagulation of RBCs) was evaluated. The overall flow rate was determined by measuring the velocity of all capillary structures visible within the frame of maximum pathology and averaging the values of all structures in that area. The RBC velocity within capillaries was calculated in µm/sec and the maximum calculable velocity was 150 µm/sec. Small venules were infrequent and most of the time not observed. When present, they represented <10% of all vessels, and did not contribute significantly to the results.

### Statistical Analyses

The two-tailed Student’s *t* test was used for data analysis. Data are expressed as mean plus the standard error of the mean (SEM). Statistical significance was set at p≤0.05.

## References

[1] Collier RJ, Young JA (2003). Anthrax toxin.. Annu Rev Cell Dev Biol.

[2] Klein F, Walker JS, Fitzpatrick DF, Lincoln RE, Mahlandt BG (1966). Pathophysiology of anthrax.. J Infect Dis.

[3] Beall FA, Dalldorf FG (1966). Pathogenesis of lethal effect of anthrax toxin in rat.. J Infect Dis.

[4] Fish DC, Klein F, Lincoln RE, Walker JS, Dobbs JP (1968). Pathophysiological changes in rat associated with anthrax toxin.. J Infect Dis.

[5] Klein F, Haines BW, Jones WI, Lincoln RE, Hodges DR (1962). Anthrax toxin: Causative agent in death of rhesus monkeys.. Science.

[6] Moayeri M, Haines D, Young HA, Leppla SH (2003). Bacillus anthracis lethal toxin induces TNF-alpha-independent hypoxia-mediated toxicity in mice.. J Clin Invest.

[7] Terra JK, Cote CK, France B, Jenkins AL, Bozue JA (2010). Cutting edge: Resistance to Bacillus anthracis infection mediated by a lethal toxin sensitive allele of Nalp1b/Nlrp1b.. J Immunol.

[8] Bradley KA, Levine SM (2010). Anthrax toxin delivers a one-two punch.. Cell Host Microbe.

[9] Bradley KA, Mogridge J, Mourez M, Collier RJ, Young JAT (2001). Identification of the cellular receptor for anthrax toxin.. Nature.

[10] Gordon VM, Klimpel KR, Arora N, Henderson MA, Leppla SH (1995). Proteolytic activation of bacterial toxins by eukaryotic cells is performed by furin and by additional cellular proteases.. Infect Immun.

[11] Ezzell JW, Abshire TG (1992). Serum protease cleavage of Bacillus anthracis protective antigen.. J Gen Microbiol 138 (Pt.

[12] Milne JC, Furlong D, Hanna PC, Wall JS, Collier RJ (1994). Anthrax protective antigen forms oligomers during intoxication of mammalian cells.. J Biol Chem.

[13] Kintzer AF, Thoren KL, Sterling HJ, Dong KC, Feld GK (2009). The protective antigen component of anthrax toxin forms functional octameric complexes.. J Mol Biol.

[14] Mogridge J, Cunningham K, Collier RJ (2002). Stoichiometry of anthrax toxin complexes.. Biochemistry.

[15] Blaustein RO, Koehler TM, Collier RJ, Finkelstein A (1989). Anthrax toxin channel-forming activity of protective antigen in planar phospholipid bilayers.. Proc Natl Acad Sci U S A.

[16] Singh Y, Klimpel KR, Goel S, Swain PK, Leppla SH (1999). Oligomerization of anthrax toxin protective antigen and binding of lethal factor during endocytic uptake into mammalian cells.. Infect Immun.

[17] Duesbery NS, Webb CP, Leppla SH, Gordon VM, Klimpel KR (1998). Proteolytic inactivation of MAP-kinase-kinase by anthrax lethal factor.. Science.

[18] Pellizzari R, Guidi-Rontani C, Vitale G, Mock M, Montecucco C (1999). Anthrax lethal factor cleaves MKK3 in macrophages and inhibits the LPS/IFN gamma-induced release of NO and TNF alpha.. FEBS Lett.

[19] Vitale G, Pellizzari R, Recchi C, Napolitani G, Mock M (1998). Anthrax lethal factor cleaves the N-terminus of MAPKKs and induces tyrosine/threonine phosphorylation of MAPKs in cultured macrophages.. Biochem Biophys Res Comm.

[20] Brodsky IE, Medzhitov R (2009). Targeting of immune signalling networks by bacterial pathogens.. Nat Cell Biol.

[21] Boyden ED, Dietrich WF (2006). Nalp1b controls mouse macrophage susceptibility to anthrax lethal toxin.. Nat Genet.

[22] Cordoba-Rodriguez R, Fang H, Lankford CS, Frucht DM (2004). Anthrax lethal toxin rapidly activates caspase-1/ICE and induces extracellular release of interleukin (IL)-1beta and IL-18.. J Biol Chem.

[23] Mariathasan S, Weiss DS, Newton K, McBride J, O’Rourke K (2006). Cryopyrin activates the inflammasome in response to toxins and ATP.. Nature.

[24] Franchi L, Eigenbrod T, Munoz-Planillo R, Nunez G (2009). The inflammasome: a caspase-1-activation platform that regulates immune responses and disease pathogenesis.. Nat Immunol.

[25] Leung BP, Culshaw S, Gracie JA, Hunter D, Canetti CA (2001). A role for IL-18 in neutrophil activation.. J Immunol.

[26] Netea MG, Fantuzzi G, Kullberg BJ, Stuyt RJ, Pulido EJ (2000). Neutralization of IL-18 reduces neutrophil tissue accumulation and protects mice against lethal Escherichia coli and Salmonella typhimurium endotoxemia.. J Immunol.

[27] Dao T, Mehal WZ, Crispe IN (1998). IL-18 augments perforin-dependent cytotoxicity of liver NK-T cells.. J Immunol.

[28] Chaix J, Tessmer MS, Hoebe K, Fuseri N, Ryffel B (2008). Cutting edge: Priming of NK cells by IL-18.. J Immunol.

[29] Schoenborn JR, Wilson CB (2007). Regulation of interferon-gamma during innate and adaptive immune responses.. Adv Immunol.

[30] Takeda K, Tsutsui H, Yoshimoto T, Adachi O, Yoshida N (1998). Defective NK cell activity and Th1 response in IL-18-deficient mice.. Immunity.

[31] Fantuzzi G, Puren AJ, Harding MW, Livingston DJ, Dinarello CA (1998). Interleukin-18 regulation of interferon gamma production and cell proliferation as shown in interleukin-1beta-converting enzyme (caspase-1)-deficient mice.. Blood.

[32] Zhu P, Xiong W, Rodgers G, Qwarnstrom EE (1998). Regulation of interleukin 1 signalling through integrin binding and actin reorganization: disparate effects on NF-kappaB and stress kinase pathways.. Biochem J 330 (Pt.

[33] Saklatvala J, Rawlinson LM, Marshall CJ, Kracht M (1993). Interleukin 1 and tumour necrosis factor activate the mitogen-activated protein (MAP) kinase kinase in cultured cells.. FEBS Lett.

[34] Dinarello CA (1996). Biologic basis for interleukin-1 in disease.. Blood.

[35] Elazar S, Gonen E, Livneh-Kol A, Rosenshine I, Shpigel NY (2010). Neutrophil recruitment in endotoxin-induced murine mastitis is strictly dependent on mammary alveolar macrophages.. Veterinary Res.

[36] McColl SR, Clark-Lewis I (1999). Inhibition of murine neutrophil recruitment in vivo by CXC chemokine receptor antagonists.. J Immunol.

[37] Terra JK, France B, Cote CK, Jenkins A, Bozue JA (2011). Allelic Variation on Murine Chromosome 11 Modifies Host Inflammatory Responses and Resistance to Bacillus anthracis.. PLoS Pathog.

[38] Welkos SL, Keener TJ, Gibbs PH (1986). Differences in susceptibility of inbred mice to Bacillus anthracis.. Infect Immun.

[39] Welkos SL, Friedlander AM (1988). Pathogenesis and genetic control of resistance to the Sterne strain of Bacillus anthracis.. Microb Pathog.

[40] Welkos SL, Trotter RW, Becker DM, Nelson GO (1989). Resistance to the Sterne strain of B. anthracis: phagocytic cell responses of resistant and susceptible mice.. Microb Pathog.

[41] Moayeri M, Crown D, Newman ZL, Okugawa S, Eckhaus M (2010). Inflammasome sensor Nlrp1b-dependent resistance to anthrax is mediated by caspase-1, IL-1 signaling and neutrophil recruitment.. PLoS Pathog.

[42] Burke JF, Miles AA (1958). The sequence of vascular events in early infective inflammation.. J Pathol Bacteriol.

[43] Wilhelm DL (1962). The mediation of increased vascular permeability in inflammation.. Pharmacol Rev.

[44] Melican K, Boekel J, Mansson LE, Sandoval RM, Tanner GA (2008). Bacterial infection-mediated mucosal signalling induces local renal ischaemia as a defence against sepsis.. Cell Microbiol.

[45] Takahashi H, Tsuda Y, Kobayashi M, Herndon DN, Suzuki F (2006). CCL2 as a trigger of manifestations of compensatory anti-inflammatory response syndrome in mice with severe systemic inflammatory response syndrome.. J Leukoc Biol.

[46] Bone RC (1996). Immunologic dissonance: a continuing evolution in our understanding of the systemic inflammatory response syndrome (SIRS) and the multiple organ dysfunction syndrome (MODS).. Ann Intern Med.

[47] Hack CE, Ogilvie AC, Eisele B, Jansen PM, Wagstaff J (1994). Initial studies on the administration of C1-esterase inhibitor to patients with septic shock or with a vascular leak syndrome induced by interleukin-2 therapy.. Prog Clin Biol Res.

[48] Terra J (2010). Characterizing the Role of Host Allelic Variation in Susceptibility to Anthrax [Thesis.. University of California, Los Angeles.]: University of California, Los Angeles.

[49] Cui XZ, Moayeri M, Li Y, Li XM, Haley M (2004). Lethality during continuous anthrax lethal toxin infusion is associated with circulatory shock but not inflammatory cytokine or nitric oxide release in rats.. Amer J Physiol - Regul Integr Comp Physiol.

[50] Bolcome RE, Sullivan SE, Zeller R, Barker AP, Collier RJ (2008). Anthrax lethal toxin induces cell death-independent permeability in zebrafish vasculature.. Proc Nat Acad Sci U S A.

[51] Stearns-Kurosawa DJ, Lupu F, Taylor FB, Kinasewitz G, Kurosawa S (2006). Sepsis and pathophysiology of anthrax in a nonhuman primate model.. Am J Pathol.

[52] Gozes Y, Moayeri M, Wiggins JF, Leppla SH (2006). Anthrax lethal toxin induces ketotifen-sensitive intradermal vascular leakage in certain inbred mice.. Infect Immun.

[53] Norman KE, Cotter MJ, Stewart JB, Abbitt KB, Ali M (2003). Combined anticoagulant and antiselectin treatments prevent lethal intravascular coagulation.. Blood.

[54] Finigan JH, Boueiz A, Wilkinson E, Damico R, Skirball J (2009). Activated protein C protects against ventilator-induced pulmonary capillary leak.. Amer J Physio - Lung Cell Mol Physiol.

[55] Vandenbroucke RE, Dejager L, Libert C (2011). The first MMP in sepsis.. EMBO Molecular Medicine.

[56] Ganter CC, Jakob SM, Takala J (2006). Pulmonary capillary pressure. A review.. Minerva Anestesiologica.

[57] McAllister RD, Singh Y, du Bois WD, Potter M, Boehm T (2003). Susceptibility to anthrax lethal toxin is controlled by three linked quantitative trait loci.. Am J Pathol.

[58] Roberts JE, Watters JW, Ballard JD, Dietrich WF (1998). Ltx1, a mouse locus that influences the susceptibility of macrophages to cytolysis caused by intoxication with Bacillus anthracis lethal factor, maps to chromosome 11.. Mol Microbiol.

[59] Levi M, van der Poll T, ten Cate H (2006). Tissue factor in infection and severe inflammation.. Semin Thromb Hemost.

[60] Levi M, van der Poll T, Schultz M (2012). New insights into pathways that determine the link between infection and thrombosis.. Neth J Med.

[61] Rao LV, Rapaport SI (1988). Activation of factor VII bound to tissue factor: a key early step in the tissue factor pathway of blood coagulation.. Proc Natl Acad Sci U S A.

[62] Ovanesov MV, Ananyeva NM, Panteleev MA, Ataullakhanov FI, Saenko EL (2005). Initiation and propagation of coagulation from tissue factor-bearing cell monolayers to plasma: initiator cells do not regulate spatial growth rate.. J Thromb Haemost.

[63] Monroe DM, Hoffman M, Roberts HR (1996). Transmission of a procoagulant signal from tissue factor-bearing cell to platelets.. Blood Coagul Fibrinolysis.

[64] Rao LV, Ngyuen M, Pendurthi UR (2004). Lethal toxin of Bacillus anthracis inhibits tissue factor expression in vascular cells.. J Thromb Haemost.

[65] Puhlmann M, Weinreich DM, Farma JM, Carroll NM, Turner EM (2005). Interleukin-1beta induced vascular permeability is dependent on induction of endothelial tissue factor (TF) activity.. J Transl Med.

[66] Dumas EK, Cox PM, Fullenwider CO, Nguyen M, Centola M (2011). Anthrax lethal toxin-induced gene expression changes in mouse lung.. Toxins.

[67] Hack CE (2003). Derangements of coagulation and fibrinolysis in infectious diseases.. Contrib Microbiol.

[68] Sun H (2006). The interaction between pathogens and the host coagulation system.. Physiology.

[69] Zugel U, Kaufmann SH (1999). Role of heat shock proteins in protection from and pathogenesis of infectious diseases.. Clin Microbiol Rev.

[70] Niatsetskaya Z, Basso M, Speer RE, McConoughey SJ, Coppola G (2010). HIF prolyl hydroxylase inhibitors prevent neuronal death induced by mitochondrial toxins: therapeutic implications for Huntington’s disease and Alzheimer’s disease.. Antioxid Redox Signal.

[71] Spinella F, Rosano L, Del Duca M, Di Castro V, Nicotra MR (2010). Endothelin-1 inhibits prolyl hydroxylase domain 2 to activate hypoxia-inducible factor-1alpha in melanoma cells.. PLoS ONE.

[72] Martinon F, Petrilli V, Mayor A, Tardivel A, Tschopp J (2006). Gout-associated uric acid crystals activate the NALP3 inflammasome.. Nature.

[73] Stroher U, West E, Bugany H, Klenk HD, Schnittler HJ (2001). Infection and activation of monocytes by Marburg and Ebola viruses.. J Virol.

[74] Kalns J, Scruggs J, Millenbaugh N, Vivekananda J, Shealy D (2002). TNF receptor 1, IL-1 receptor, and iNOS genetic knockout mice are not protected from anthrax infection.. Biochem Biophys Res Commun.

[75] Shikama Y, Kuroishi T, Nagai Y, Iwakura Y, Shimauchi H (2011). Muramyldipeptide augments the actions of lipopolysaccharide in mice by stimulating macrophages to produce pro-IL-1beta and by down-regulation of the suppressor of cytokine signaling 1 (SOCS1).. Innate Immun.

[76] Kawa K, Tsutsui H, Uchiyama R, Kato J, Matsui K (2010). IFN-gamma is a master regulator of endotoxin shock syndrome in mice primed with heat-killed Propionibacterium acnes.. Internat Immunol.

[77] Klein F, Lincoln RE, Mahlandt BG, Dodds JP, Walker JS (1967). Effect of temperature and drug therapy on anthrax intoxications.. Proc Soc Exp Biol Med.

[78] Jin Y, Mailloux CM, Gowan K, Riccardi SL, LaBerge G (2007). NALP1 in vitiligo-associated multiple autoimmune disease.. New Engl J Med.

[79] Lev-Sagie A, Prus D, Linhares IM, Lavy Y, Ledger WJ (2009). Polymorphism in a gene coding for the inflammasome component NALP3 and recurrent vulvovaginal candidiasis in women with vulvar vestibulitis syndrome.. Amer J Obst Gyn 200: 303 e301–306.

[80] Sutherland AM, Walley KR (2009). Bench-to-bedside review: Association of genetic variation with sepsis.. Crit Care.

[81] Iakoubova OA, Olsson CL, Dains KM, Ross DA, Andalibi A (2001). Genome-tagged mice (GTM): two sets of genome-wide congenic strains.. Genomics.

[82] Davis RC, Jin A, Rosales M, Yu S, Xia X (2007). A genome-wide set of congenic mouse strains derived from CAST/Ei on a C57BL/6 background.. Genomics.

[83] Culley NC, Pinson DM, Chakrabarty A, Mayo MS, Levine SM (2005). Pathophysiological manifestations in mice exposed to anthrax lethal toxin.. Infect Immun.

[84] Wood JG, Johnson JS, Mattioli LF, Gonzalez NC (2000). Systemic hypoxia increases leukocyte emigration and vascular permeability in conscious rats.. J Appl Physiol.

[85] Steiner DR, Gonzalez NC, Wood JG (2001). Leukotriene B(4) promotes reactive oxidant generation and leukocyte adherence during acute hypoxia.. J Appl Physiol.

[86] McLoughlin RM, Witowski J, Robson RL, Wilkinson TS, Hurst SM (2003). Interplay between IFN-gamma and IL-6 signaling governs neutrophil trafficking and apoptosis during acute inflammation.. J Clin Invest.

[87] Xin L, Li Y, Soong L (2007). Role of interleukin-1beta in activating the CD11c(high) CD45RB- dendritic cell subset and priming Leishmania amazonensis-specific CD4+ T cells in vitro and in vivo.. Infect Immun.

[88] Björkbacka H, Kunjathoor VV, Moore KJ, Koehn S, Ordija CM (2004). Reduced atherosclerosis in MyD88-null mice links elevated serum cholesterol levels to activation of innate immunity signaling pathways.. Nat Med.

[89] Sutterwala FS, Ogura Y, Szczepanik M, Lara-Tejero M, Lichtenberger GS (2006). Critical role for NALP3/CIAS1/Cryopyrin in innate and adaptive immunity through its regulation of caspase-1.. Immunity.

[90] Gray H (1918). Anatomy of the Human Body; Lewis WH, editor. Philadelphia: Lea & Febiger. Fig. 535.. http://en.wikipedia.org/wiki/File:Gray535.png.

[91] Fleming KK, Bovaird JA, Mosier MC, Emerson MR, LeVine SM (2005). Statistical analysis of data from studies on experimental autoimmune encephalomyelitis.. J Neuroimmunol.

